# Mediating effect of psychological capital on the relationship between work engagement and perceived professional benefits among nursing interns: a cross-sectional study

**DOI:** 10.3389/fmed.2026.1802670

**Published:** 2026-06-08

**Authors:** Sixing Liu, Haitang Liu, Fengying Lin, Lihua Chen, Yuanfeng Lv, Fan Wei, Chaoluan Rao, Xiaojing Chen, Caihong Xue, Minxiang Li

**Affiliations:** 1 Department of Nursing, The Second Affiliated Hospital of Hainan Medical University, Haikou, Hainan, China; 2 School of Nursing, Hainan Medical University, Haikou, Hainan, China; 3 Department of Hepatobiliary and Pancreatic Surgery, The Second Affiliated Hospital of Hainan Medical University, Haikou, Hainan, China

**Keywords:** mediation analysis, nursing interns, perceived professional benefits, psychological capital, work engagement

## Abstract

**Background:**

Nursing interns represent a vital reserve for the nursing workforce, and their perceived professional benefits are critical to workforce stability. However, the mechanisms linking their work engagement, psychological capital, and perceived professional benefits remain underexplored in this population.

**Objectives:**

This study aims to examine the relationships among nursing interns’ perceived professional benefits, work engagement, and psychological capital and to further explore the mediating role of psychological capital.

**Methods:**

In this cross-sectional study, 363 nursing interns were recruited from six Grade A tertiary hospitals in Hainan Province, China. Data were collected using the Demographic Information Questionnaire, the Nurses’ Perceived Professional Benefit Questionnaire, the Utrecht Work Engagement Scale-9, and the Psychological Capital Questionnaire. A structural equation model was constructed using AMOS 24.0 software to test the mediating role of psychological capital.

**Results:**

The nurses’ work engagement score was 30.17 ± 7.83, their psychological capital score was 84.66 ± 13.99, and their perceived professional benefits score was 67.14 ± 8.52. Pearson correlation analysis revealed a positive correlation between nursing interns’ work engagement and perceived professional benefits (*r* = 0.480, *P* < 0.001). Work engagement was positively correlated with psychological capital (*r* = 0.480, *P* < 0.001). Psychological capital was positively correlated with perceived professional benefits (*r* = 0.506, *P* < 0.001). Furthermore, psychological capital significantly mediated the association between work engagement and perceived professional benefits, with the indirect effect accounting for 36.04% of the total effect.

**Conclusion:**

Within the internship management system, clinical nursing administrators should work together to enhance nursing interns’ work engagement and cultivate their psychological capital, thereby increasing their perceived professional benefits. This will strengthen their willingness to pursue a career in nursing and lay the foundation for a stable nursing workforce.

## 1 Introduction

The shortage of nursing personnel is a global challenge, and the World Health Organization estimates that the global nurse shortage will reach 5.7 million by 2030 (1). The situation in China is even more severe: by the end of 2023, there were only 4 registered nurses per 1,000 people, far below the average in developed countries (2). Although the number of nursing graduates continues to grow, this has not alleviated the shortage (3), largely because of the high attrition rate among new nurses (2). The career stability of new nurses depends largely on their adaptation during the clinical internship phase and the formation of their professional identity (4). Clinical internships serve as a transition from nursing interns to prenurses and constitute a critical period for the development of professional attitudes and values (5). However, during this phase, nursing interns often face multiple pressures, including insufficient knowledge, unfamiliarity with the environment and patient histories, excessive workloads, fear of procedural errors, and difficulties in using medical equipment, potentially resulting in excessive resource depletion (6).

To elucidate the patterns of dynamic change in individual resources among nursing interns in this context, conservation of resources (COR) theory provides important theoretical support and has been widely applied in research on the mental health and occupational adaptation of nursing staff (7). COR theory posits that individuals in stressful situations tend to acquire, conserve, and maintain their resources to cope with challenges (8, 9). Additionally, this theory also proposes resource gain and loss spirals, whereby resource loss tends to trigger a chain reaction, creating a downward spiral, while the accumulation of resources enhances an individual’s capacity for resource investment, thereby enabling the acquisition of more resources and creating an upward spiral (8, 9).

With the rise of positive psychology, nursing scholars have increasingly adopted a positive perspective to explore nurses’ professional attitudes, with a particular focus on perceived professional benefits (10). Perceived professional benefits refer to nurses’ subjective perception of the positive gains derived from their work and serve as a cognitive adaptation strategy for positive professional emotions (11). Research has indicated that perceived professional benefits help nursing interns develop positive perceptions of their work, gain growth experiences, and strengthen their commitment to the profession (12, 13). Furthermore, studies have shown that perceived professional benefits are positively correlated with both work engagement and psychological capital (14, 15). On the one hand, high levels of work engagement enable nursing staff to experience greater pleasure and satisfaction, which helps them maintain positive professional attitudes and, in turn, positively enhances their perceived professional benefits (14). On the other hand, psychological capital, as a vital internal resource, helps individuals develop an adaptive mindset and enhance their resilience, encouraging them to view their profession from a more positive perspective and thereby experience stronger perceived professional benefits (15).

Furthermore, work engagement and psychological capital form a resource gain spiral (16). Research has indicated that work engagement has a positive predictive effect on psychological capital (17). This positive psychological energy, generated by high work engagement, serves as a motivational resource that drives the cultivation and accumulation of an individual’s psychological capital (17). Consequently, work engagement, as a form of resource investment, provides individuals with positive career experiences, whereas psychological capital, as an internal resource, strengthens individuals’ ability to cope with workplace stress and their perception of value.

However, previous studies have focused primarily on pairwise relationships among variables such as work engagement and psychological capital (18), work engagement and perceived professional benefits (19), and psychological capital and perceived professional benefits (15). However, the underlying mechanisms among these three variables have not yet been clarified among nursing interns. Therefore, on the basis of COR theory, this study examines nursing interns to investigate the current status and relationships among their work engagement, psychological capital, and perceived professional benefits and to explore the mediating role of psychological capital between work engagement and perceived professional benefits. On this basis, the study proposes the following hypotheses:

H1: Nursing interns’ work engagement is positively correlated with their perceived professional benefits.

H2: Nursing interns’ work engagement is positively correlated with psychological capital.

H3: Nursing interns’ psychological capital is positively correlated with perceived professional benefits.

H4: Psychological capital mediates the relationship between nursing interns’ work engagement and perceived professional benefits.

## 2 Materials and methods

### 2.1 Design and setting

This cross-sectional quantitative study was conducted in accordance with the Strengthening the Reporting of Observational Studies in Epidemiology (STROBE) guidelines.

### 2.2 Study participants

From October 2024 to January 2025, nursing interns were recruited through convenience sampling from six Grade A tertiary hospitals in Hainan Province, China. Participants included both specialized and undergraduate program students. The inclusion criteria were as follows: (a) nursing interns in specialized or undergraduate programs who had completed all the required coursework; (b) an internship duration of ≥6 months; and (c) nursing interns who provided informed consent and voluntarily agreed to participate in this study. The exclusion criteria were as follows: (a) nursing interns with severe mental or psychological issues that prevented them from cooperating with the study; and (b) nursing interns who were sent back to their schools by the hospital because of violations of internship regulations.

The sample size was estimated on the basis of the requirements of structural equation modeling (SEM). In this study, the dimension scores of the scales were used as observed indicators of the latent variables. The model included three latent variables (work engagement, psychological capital, and perceived professional benefits) and 12 observed variables (three dimensions of work engagement, four dimensions of psychological capital, and five dimensions of perceived professional benefits). According to Kline (20), the sample size for SEM should be at least 10 times the number of free parameters in the model and no fewer than 200 cases. This model contained a total of 27 free parameters, including factor loadings, measurement error variances, structural path coefficients, and residual variances of latent variables. Thus, the minimum required sample size was 270. To account for a 20% rate of invalid questionnaires, at least 338 questionnaires needed to be distributed. A total of 380 questionnaires were collected. After the exclusion of invalid responses, 363 valid questionnaires were retained, yielding an effective response rate of 95.53%. The sample size met the requirements for SEM analysis.

### 2.3 Measures

#### 2.3.1 Demographic information questionnaire

The Demographic Information Questionnaire was developed by the researchers on the basis of the research objectives and literature review. It included items on gender, education level, place of origin, only child status, experience as a student leader, monthly per capita household income, having relatives or friends in nursing, reasons for choosing nursing major, and postgraduation employment choice.

#### 2.3.2 Nurses’ Perceived Professional Benefit Questionnaire (NPPBQ)

The NPPBQ was developed by Hu et al. (21) and later simplified by Hu et al. (22) through expert consultation and interviews. The questionnaire consists of 17 items across five dimensions: positive occupational perception (three items); good nurse-patient relationship (four items); recognition from family, relatives, and friends (three items); sense of belonging to a team (three items); and self-growth (four items). Each item uses a five-point Likert scale ranging from 1 (strongly disagree) to 5 (strongly agree). Total scores range from 17 to 85, with higher scores indicating greater perceived professional benefits. In this study, the overall Cronbach’s α was 0.960, and the Cronbach’s α values of the dimensions were 0.823, 0.848, 0.800, 0.815, and 0.841, demonstrating good internal consistency reliability.

#### 2.3.3 Utrecht Work Engagement Scale-9 (UWES-9)

The UWES-9, developed by Schaufeli et al. (23) and translated and revised by Zhang et al. (24), measures work engagement. The scale consists of nine items across three dimensions: vigor (three items), dedication (three items), and absorption (three items), Each item is rated on a seven-point Likert scale ranging from 0 (never) to 6 (always). Total scores range from 0 to 54, with higher scores indicating greater work engagement. In this study, the overall Cronbach’s α was 0.973, and the Cronbach’s α values of the dimensions were 0.919, 0.924, and 0.923, demonstrating good internal consistency reliability.

#### 2.3.4 Psychological Capital Questionnaire (PCQ)

The PCQ was developed by Luthans et al. (25) and translated by Luo et al. (26) to assess psychological capital. The Chinese version comprises 20 items across four dimensions: self-efficacy (six items), hope (six items), resilience (five items), and optimism (three items). Each item is rated on a six-point Likert scale scored from 1 (strongly disagree) to 6 (strongly agree). Total scores range from 20 to 120, with higher scores indicating greater psychological capital. In this study, the overall Cronbach’s α was 0.982, and the Cronbach’s α values of the dimensions were 0.940, 0.944, 0.929, and 0.890, demonstrating good internal consistency reliability.

### 2.4 Data collection

Prior to the start of the study, the corresponding author contacted the nursing departments of the hospitals where the survey was to be conducted and explained the background, objectives, significance, target population, and data collection methods of the study. After approval was obtained from the head of the nursing department, the department distributed the questionnaires to the nursing interns who met the inclusion criteria. The questionnaires were distributed via an online survey platform (Wenjuanxing^1^). The questionnaire included a demographic information questionnaire, the NPPBQ, the UWES-9, and the PCQ. Upon accessing the questionnaire, the nursing interns were presented with a detailed study introduction, instructions for completion, and an informed consent form on the first page. After they read these, the nursing interns had to click “Agree to Participate” to proceed to the main questionnaire; clicking “Disagree to Participate” terminated the process and resulted in the questionnaire being marked as invalid. To prevent duplicate responses, the survey was configured to allow only one response per IP address. Additionally, all the questions were marked as required, and the survey could not be submitted until all the questions were completed. After all the data were collected, two researchers conducted a thorough review, excluding surveys with completion times less than 3 min and those in which all the answers selected were identical.

### 2.5 Statistical analysis

The data were analyzed using SPSS 22.0 software. Categorical data are presented as frequencies and percentages. Continuous data are described as the means ± standard deviations, and normality was tested using skewness and kurtosis. When the absolute value of skewness was <2 and the absolute value of kurtosis was <7, the data were considered to follow a normal distribution (27). All the continuous variables in this study followed a normality distribution. On this basis, Pearson correlation analysis was used to investigate the relationships among work engagement, psychological capital, and perceived professional benefits. Furthermore, AMOS 24.0 software was used to construct an SEM, and the mediating effects were tested using a bias-corrected non-parametric bootstrap method (with 5,000 repetitions). A *P*-value < 0.05 was considered to indicate statistical significance.

## 3 Results

### 3.1 Demographic characteristics of participants

This study included 363 nursing interns. Among them, 336 were female (92.56%), and 27 were male (7.44%). In terms of educational background, the majority held bachelor’s degrees (298, or 82.09%), while 65 held associate degrees (17.91%). With respect to place of origin, 223 students (61.43%) were from rural areas, while 140 (38.57%) were from urban areas. Additionally, the majority were not only children, totaling 299 (82.37%), while 64 (17.63%) were only children. Further detailed demographic data are presented in Table 1.

**TABLE 1:** Demographic characteristics of the study participants (*N* = 363).

Variables	Categories	*n*	%
Experience as a student leader	Yes	165	45.45
	No	198	54.55
Monthly per capita household income	<3,000 CNY	65	17.91
	3,001–5,000 CNY	169	46.56
	5,001–10,000 CNY	94	25.90
	>10,001 CNY	35	9.64
Relatives or friends in nursing	Yes	139	38.29
	No	224	61.71
Reasons for choosing nursing major	Personal interest	47	12.95
	Employment prospects	136	37.47
	Parent or relative choice	95	26.17
	College entrance exam adjustment	85	23.42
Postgraduation employment choice	Continue nursing work	225	61.98
	Pursue nursing graduate studies	81	22.31
	Pursue non-nursing graduate studies	9	2.48
	Change profession	48	13.22

Owing to rounding, the sum of the percentages may not equal 100.00%.

### 3.2 Common method bias test

Common method bias was assessed using Harman’s single-factor test. All the items from the three scales (work engagement, psychological capital, and perceived professional benefits) were subjected to unrotated exploratory factor analysis. The first principal component before rotation explained 48.241% of the variance, which was below the 50% threshold proposed by Podsakoff and Organ (28), indicating that no serious common method bias was present in this study.

### 3.3 Scores of the study variables

The total score for nursing interns’ work engagement was 30.17 ± 7.83, the total score for psychological capital was 84.66 ± 13.99, and the total score for perceived professional benefits was 67.14 ± 8.52. Refer to Table 2 for comprehensive details.

**TABLE 2:** The scores for perceived professional benefits, work engagement, and psychological capital (*N* = 363).

Variables	M ± SD	Scoring range
Work engagement	30.17 ± 7.83	0 ∼ 54
Vigor	3.34 ± 0.88	0 ∼ 18
Dedication	3.35 ± 0.90	0 ∼ 18
Absorption	3.37 ± 0.90	0 ∼ 18
Psychological capital	84.66 ± 13.99	20 ∼ 120
Self-efficacy	4.22 ± 0.70	6 ∼ 36
Hope	4.23 ± 0.72	6 ∼ 36
Resilience	4.25 ± 0.71	5 ∼ 30
Optimism	4.21 ± 0.74	3 ∼ 18
Perceived professional benefits	67.14 ± 8.52	17 ∼ 85
Positive occupational perception	3.95 ± 0.55	3 ∼ 15
Good nurse-patient relationship	3.96 ± 0.53	4 ∼ 20
Recognition from family, relatives, and friends	3.95 ± 0.55	3 ∼ 15
Sense of belonging to a team	3.95 ± 0.55	3 ∼ 15
Self-growth	3.94 ± 0.53	4 ∼ 20

### 3.4 Correlation analysis of the study variables

The results of the Pearson correlation analysis indicate that nursing interns’ work engagement is positively correlated with their perceived professional benefits (*r* = 0.480, *P* < 0.001). Work engagement is positively correlated with psychological capital (*r* = 0.480, *P* < 0.001). Psychological capital is positively correlated with perceived professional benefits (*r* = 0.506, *P* < 0.001). Refer to Table 3 for comprehensive details.

**TABLE 3:** Correlation analysis of perceived professional benefits, work engagement, and psychological capital (*N* = 363).

Variables	Perceived professional benefits	Work engagement	Psychological capital
Perceived professional benefits	1	–	–
Work engagement	0.480***	1	–
Psychological capital	0.506***	0.480***	1

***Correlation is significant at the 0.001 level (two-tailed).

### 3.5 Mediating effect of psychological capital between work engagement and perceived professional benefits

On the basis of the results of the correlation analysis, SEM was used to test the mediating effect, with work engagement as the independent variable, psychological capital as the mediating variable, and perceived professional benefits as the dependent variable. The model was refined and fitted using the maximum likelihood method. The model fit results indicate that the adjusted model has a good fit across all the metrics: *χ*^2^/*df* = 1.268 (<3), RFI = 0.987 (>0.9), CFI = 0.998 (>0.9), GFI = 0.971 (>0.9), IFI = 0.998 (>0.9), TLI = 0.997 (>0.9), NFI = 0.990 (>0.9), AGFI = 0.956 (>0.9), and RMSEA = 0.027 (<0.08).

**FIGURE 1:**
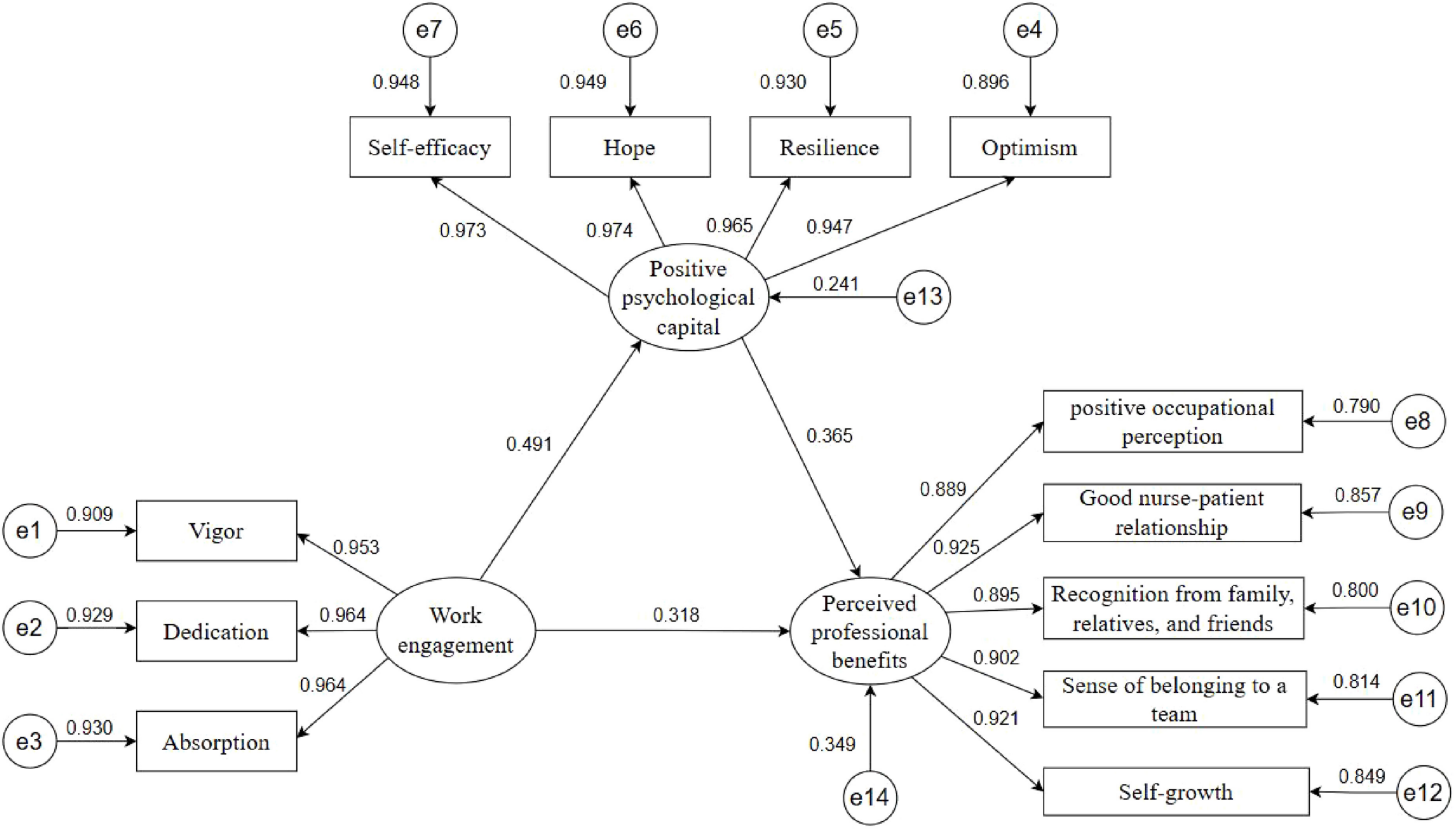
Structural equation modeling (SEM) of the indirect effect of psychological capital on the relationship between work engagement and perceived professional benefits among nursing interns. e1–e12 are measurement errors of the observed indicators; e13 and e14 are disturbance terms (residual variances) of the endogenous latent variables. All values are standardized.

Path analysis revealed that the direct path coefficient between work engagement and perceived professional benefits was significant (β = 0.318, *P* < 0.001). In addition, significant positive associations were found between work engagement and psychological capital (β = 0.491, *P* < 0.001), and between psychological capital and perceived professional benefits (β = 0.365, *P* < 0.001). As endogenous latent variables, psychological capital and perceived professional benefits had R^2^ values of 0.759 and 0.651, respectively, with corresponding disturbance variances of 0.241 (e13) and 0.349 (e14). Further analysis revealed a significant indirect path of psychological capital in the relationship between work engagement and perceived professional benefits, with an indirect path coefficient of 0.102, accounting for 36.04% of the total effect. Additionally, testing this indirect path using a bias-corrected non-parametric bootstrap method (with 5,000 repetitions) revealed that the 95% CI did not include 0, indicating that the indirect path is statistically significant. Refer to Figure 1 and Table 4 for comprehensive details.

**TABLE 4:** Results of the test for the indirect effect of psychological capital on the relationship between work engagement and perceived professional benefits among nursing interns (*N* = 363).

Effect	Estimate	SE	LLCI	ULCI	*P*	Proportion mediated (%)
Indirect effect	0.102	0.025	0.058	0.157	<0.001	36.04
Direct effect	0.181	0.044	0.098	0.270	<0.001	63.96
Total effect	0.283	0.036	0.214	0.358	<0.001	–

## 4 Discussion

The findings of this study support the hypotheses that perceived professional benefits are positively correlated with both work engagement and psychological capital, and that psychological capital mediates the relationship between nursing interns’ work engagement and their perceived professional benefits.

The results indicate that nursing interns’ scores for perceived professional benefits were in the upper-middle range, which is consistent with the findings of Liu et al. (29) in their survey of Chinese nursing interns. Among the various dimensions, the “good nurse-patient relationship” dimension had the highest mean score. This finding suggest that positive nurse-patient interactions contribute to enhancing nursing interns’ perceived professional benefits and sense of accomplishment (30). In contrast, the scores for the “self-growth” dimension were relatively low. This may be attributed to nursing interns’ limited opportunities for clinical practice and a lack of targeted growth guidance and clear feedback on skill progression. As a result, it becomes difficult for them to clearly perceive improvements in their professional competence (31).

Furthermore, the scores for perceived professional benefits among nursing interns were slightly lower than those reported by Chen et al. (32), in their survey of new nurses. This discrepancy may stem from the fact that compared with new nurses, nursing interns are still in the early stages of professional socialization. Their role identity may be less defined, their clinical experience and coping abilities relatively limited, and their internalization of professional values still shallow, resulting in weaker perceived professional benefits (33). Therefore, it is recommended that departments establish a progressive list of clinical tasks to clarify weekly practical skill objectives for nursing interns (34). Moreover, process-oriented feedback should be strengthened, with clinical instructors documenting nursing interns’ specific progress and areas for improvement on a weekly basis, enabling them to clearly track their own growth trajectory (35).

Nursing interns’ scores for work engagement were in the upper-middle range. Among the dimensions, the “absorption” dimension received the highest score. This may be related to the task-oriented nature of clinical nursing work (36). Nursing procedures require a high degree of precision, which prompts nursing interns to maintain intense concentration during clinical practice and thereby demonstrate a strong state of focused engagement (36). Scores on the “vitality” dimension were relatively low, which may be closely related to the sustained high-intensity workload during clinical rotation (37). Nursing interns not only had to complete theoretical and clinical tasks but also frequently faced night shift rotations; some also had to prepare for entrance exams in their spare time (38). Under the pressure of these multiple tasks, they found it difficult to maintain high levels of work vitality (38). Jin et al. (39) reported that high-intensity workloads can lead to excessive depletion of individual resources, resulting in fatigue and reduced vitality. Consequently, it is recommended that clinical nursing administrators allocate work tasks reasonably and schedule night shifts appropriately to prevent nursing interns from remaining in a high-intensity work state for extended periods (36). Additionally, training in stress management and mindfulness-based stress reduction can be provided to nursing interns to help them develop positive coping strategies and enhance their self-regulation abilities (40).

The psychological capital scores of the nursing interns were in the upper-middle range but were slightly lower than those reported by Gong et al. (41). This discrepancy may be related to the degree of autonomy in their choice of major. In this study, nearly half (49.59%) of the nursing interns had their major decided by parents or relatives or were admitted through college entrance exam reallocation, indicating a certain degree of passivity in their choice of major. In contrast, Gong et al. (41) reported that 75.30% of nursing interns chose a nursing major on the basis of personal preference, and they tended to have stronger intrinsic motivation and professional identity. This aligns with the view of Liu et al. (42), who noted that nursing interns who selected nursing as their first-choice major were often driven by intrinsic interest, held higher expectations for their career development, and were more confident in actively overcoming difficulties when faced with challenges.

From the perspective of COR theory, nursing interns who make a passive choice may have insufficient initial professional identity and relatively weak psychological resources. When facing the pressures of clinical practice, they are more likely to fall into a resource loss spiral, leading to burnout and self-doubt, which in turn has a downward pull on their psychological capital levels. Therefore, schools can implement early career interventions targeting professional identity for nursing interns who passively choose their major to help them replenish their initial psychological resources (41). Furthermore, during the internship phase, clinical instructors should proactively identify stress signals and signs of resource depletion among these nursing interns, promptly adjust their workload, and provide positive feedback.

This study revealed that nursing interns’ perceived professional benefits were positively correlated with both work engagement and psychological capital. On the one hand, high levels of work engagement enable nursing interns to more easily improve their skills and gain patient recognition during clinical practice. The resulting sense of accomplishment helps reinforce their professional identity, thereby enhancing their perceived professional benefits (43, 44). On the other hand, psychological capital, as a positive psychological resource, can help nursing interns maintain a positive mindset and perceive professional growth amid challenges by buffering clinical stress, thereby enhancing their perceived professional benefits (45). Additionally, this study revealed a positive correlation between work engagement and psychological capital. This may stem from the abundant practical opportunities and positive feedback resulting from high levels of work engagement, which effectively enhance nursing interns’ self-efficacy and consequently promote the accumulation of psychological capital (45). These findings suggest that clinical nursing administrators could establish a systematic positive feedback mechanism to improve nursing interns’ positive psychological state (46). For example, recognition could be given during monthly internship summaries and end-of-rotation meetings for improvements in skills, positive evaluations by clinical instructors, and patient satisfaction.

The results of this study indicate that psychological capital indirectly affects the relationship between nursing interns’ work engagement and their perceived professional benefits, accounting for 36.04% of the total effect. These findings suggest that work engagement is positively associated with perceived professional benefits through the indirect pathway of psychological capital. These findings align with COR theory (8), which posits that under stressful conditions, individuals engage in resource investment behaviors to acquire new resources and compensate for existing losses, thereby maintaining a dynamic balance of psychological resources and avoiding a resource loss spiral.

Clinical internships are a critical stage in the professional socialization process of nursing interns in China (47). In the high-pressure hospital environment, nursing interns must translate theoretical knowledge into clinical practice, familiarize themselves with nursing workflows, continuously improve their clinical skills and reasoning, and transition from students to prelicensed nurses in real-world work settings (48). However, this process is accompanied by multiple challenges. Nursing interns often face multiple stressors, including heavy workloads, interpersonal pressures, skill deficiencies, difficulties in role transition, and uncertainty regarding future career development (49). If these stressors are not effectively managed, nursing interns may experience psychological imbalance, leading to the continuous depletion of psychological resources and, consequently, diminished perceived professional benefits.

In this context, work engagement can be viewed as a form of resource investment by nursing interns when they face clinical stress. Work engagement refers to a sustained, positive psychological state exhibited by individuals in the workplace that is manifested as a high level of commitment to one’s profession (44). This state of vitality and focus can channel positive psychological energy, forming a motivational resource that effectively promotes the cultivation and accumulation of psychological capital (17). Psychological capital is also a positive psychological resource that not only helps stimulate nursing interns’ intrinsic motivation to actively integrate into the clinical environment and pursue self-growth but also enhances their psychological resilience when they face professional challenges. It encourages them to view the nursing profession from a more positive perspective and thereby enables them to experience stronger perceived professional benefits (15). Consequently, hospitals should establish psychological support centers to provide nursing interns with individualized guidance on stress identification and emotional regulation. Additionally, psychological capital assessment and cultivation should be integrated into the internship support system. Through methods such as group counseling or peer support, nursing interns can be assisted in building a buffer of positive psychological resources (41).

### 4.1 Study strengths and limitations

Using COR theory as a framework, this study employed a cross-sectional design to analyze the relationships among nursing interns’ work engagement, psychological capital, and perceived professional benefits. It clarified the mediating role of psychological capital in this relationship, thereby addressing the limitations of previous studies, which often examined only pairwise relationships between variables. Additionally, this study provides clinical nursing administrators with strategic recommendations for enhancing nursing interns’ perceived professional benefits, which is highly important for strengthening their professional identity and consolidating the nursing workforce.

This study has several limitations. First, as a cross-sectional study, it can reflect only the correlation between nursing interns’ work engagement, psychological capital, and perceived professional benefits at a specific point in time; it cannot objectively determine the causal sequence or causal relationships among the variables. Future research could employ longitudinal or experimental designs to further validate the causal pathways between these variables. Second, this study employed convenience sampling, selecting only the nursing specialty and undergraduate interns from six Grade A tertiary hospitals in Hainan Province as the study population. This sampling method introduces selection bias, and the limited geographical scope, hospital tier, and homogeneity of the study population somewhat restrict the representativeness of the findings. Third, all the data in this study were self-reported by the participants and are therefore susceptible to the influence of subjective perceptions. Fourth, the study did not account for certain potential confounding factors. Additionally, the sample source was concentrated, and the lack of a multicenter, stratified, randomized sampling design may have introduced sample distribution bias and unmeasured confounding, making it difficult to fully reflect the actual status of nursing interns across different regions and educational levels. Future studies could adopt a multicenter, large-sample, randomized sampling design to expand the geographical and institutional scope of the survey, strictly control for confounding factors, and optimize the research design. In addition, future studies should combine multiple survey methods to reduce the bias associated with self-reports alone, thereby further deepening the exploration of the relationships among these three factors.

## 5 Conclusion

This study revealed that nursing interns’ perceived professional benefits and work engagement are positively correlated with their level of psychological capital. Additionally, psychological capital mediates the relationship between work engagement and perceived professional benefits. Therefore, it is recommended that clinical nursing administrators enhance nursing interns’ work engagement by implementing measures such as establishing progressive task lists, setting up a system of process-oriented feedback, and appropriately allocating work tasks. Furthermore, psychological capital assessment and cultivation should be integrated into the internship support system, with systematic training provided in areas such as stress management, self-efficacy, and optimism. Special attention should be given to nursing interns who passively choose nursing as their major; their risk of resource depletion should be identified early and addressed promptly to help them build a positive psychological buffer, strengthen their confidence in coping with professional challenges, and thereby enhance their perceived professional benefits.

## Data Availability

The raw data supporting the conclusions of this article will be made available by the authors, without undue reservation.

## Footnotes

1
https://www.wjx.cn
